# Letter to the Editor: The effects of ChatGPT on patient education of knee osteoarthritis: a preliminary study of 60 cases

**DOI:** 10.1097/JS9.0000000000002706

**Published:** 2025-06-19

**Authors:** Peng Tang, Yongli Zhao, Jun Wang

**Affiliations:** Department of Pharmacology, Chengdu Integrated TCM & Western Medicine Hospital, Chengdu 610000, China

*Dear Editor*,

We read with great interest the article recently published in your journal on the use of ChatGPT to enhance patient education^[1]^. The authors conducted a prospective study comparing three approaches to improving patients’ knowledge acquisition: face-to-face education by physicians, unstructured interaction with ChatGPT, and structured dialogue with ChatGPT based on preset questions. Given the increasing focus on AI-assisted preoperative education and its potential to deliver high-quality medical information while reducing the burden on healthcare providers^[2,3]^, this study is a timely and thought-provoking exploration of AI’s potential in health education.

However, we believe there are several aspects of the study design, evaluation system, and generalizability of the conclusions that warrant further discussion:

- **Is ChatGPT’s “education” truly based on active patient learning?**

The authors attributed the poor performance of the free-interaction group to a lack of structured prompts, but this highlights a broader issue: ChatGPT, when used without professional guidance, may not be capable of delivering effective health education. The assumption that ChatGPT can serve as a universally accessible educational tool might be overly optimistic, and this limitation was not sufficiently addressed in the discussion, potentially leading to an overestimation of AI’s educational potential.

- **Subjective bias in primary endpoint assessment**

The study relied on a visual analog scale (VAS) to assess patients’ knowledge acquisition, as evaluated by independent physicians. However, no quantitative criteria or evaluation dimensions were provided. Moreover, VAS scoring is highly sensitive to communication and expressive abilities, which may obscure a true assessment of patients’ knowledge. The lack of more objective and reproducible tools, such as standardized knowledge questionnaires, limits the reliability of the outcome.

- **Lack of standardized framework for evaluating ChatGPT’s response quality**

The authors conducted a “quality assessment” of ChatGPT’s responses, but the scoring methodology was not described in detail and did not incorporate widely accepted tools for evaluating medical information quality (e.g., DISCERN, JAMA benchmark). As a result, the explanatory power and credibility of these findings are limited.

- **Small sample size and lack of multi-center validation**

The study included only 60 participants from a single center, which raises concerns about the influence of local educational and cultural backgrounds on the results. Given that AI-generated responses are highly context-dependent, the absence of multi-center and multilingual validation limits the generalizability of the conclusions.

- **Lack of assessment of behavioral or prognostic impact**

While knowledge acquisition is important, the ultimate goal of patient education is to promote behavioral change and improve clinical outcomes. The study did not explore whether the different educational approaches led to changes in diet, exercise, or medication adherence. Without such follow-up, the “effectiveness” of ChatGPT-based education remains superficial.

In summary, while this study is a valuable exploratory attempt, its methodology and interpretation warrant a more cautious approach. We recommend that future studies focus on: (1) establishing objective frameworks to evaluate educational outcomes; (2) conducting longitudinal follow-up to assess behavioral changes after ChatGPT-based education; and (3) validating AI education tools in diverse linguistic and cultural settings to ensure their safe and effective clinical implementation.

## Data Availability

Not applicable.

## Research registration unique identifying number (UIN)

There are no conflicts of interest.
